# An Interactive Online App for Predicting Diabetes via Machine Learning from Environment-Polluting Chemical Exposure Data

**DOI:** 10.3390/ijerph19105800

**Published:** 2022-05-10

**Authors:** Rosy Oh, Hong Kyu Lee, Youngmi Kim Pak, Man-Suk Oh

**Affiliations:** 1 Department of Mathematics, Korea Military Academy, Seoul 01805, Korea; rosy.oh5@gmail.com; 2 Department of Internal Medicine, College of Medicine, Seoul National University, Seoul 03080, Korea; hongkyu414@gmail.com; 3 Department of Physiology, College of Medicine, Kyung Hee University, Seoul 02447, Korea; 4 Department of Statistics, Ewha Womans University, Seoul 03760, Korea

**Keywords:** diabetes mellitus, glucose intolerance, machine learning, Bayesian network, environmental pollutants

## Abstract

The early prediction and identification of risk factors for diabetes may prevent or delay diabetes progression. In this study, we developed an interactive online application that provides the predictive probabilities of prediabetes and diabetes in 4 years based on a Bayesian network (BN) classifier, which is an interpretable machine learning technique. The BN was trained using a dataset from the Ansung cohort of the Korean Genome and Epidemiological Study (KoGES) in 2008, with a follow-up in 2012. The dataset contained not only traditional risk factors (current diabetes status, sex, age, etc.) for future diabetes, but it also contained serum biomarkers, which quantified the individual level of exposure to environment-polluting chemicals (EPC). Based on accuracy and the area under the curve (AUC), a tree-augmented BN with 11 variables derived from feature selection was used as our prediction model. The online application that implemented our BN prediction system provided a tool that performs customized diabetes prediction and allows users to simulate the effects of controlling risk factors for the future development of diabetes. The prediction results of our method demonstrated that the EPC biomarkers had interactive effects on diabetes progression and that the use of the EPC biomarkers contributed to a substantial improvement in prediction performance.

## 1. Introduction

Diabetes is slowly rising across the globe. The prevalence of diabetes has been steadily increasing over the past few decades [1,2,3]. About 422 million people live with diabetes as of 2020 [4]. By 2045, more than 700 million people are expected to have diabetes [1]. Diabetes mellitus (DM) is associated with a wide range of serious health complications that affect the renal, neurological, cardiac, and vascular systems, and it has a major impact on overall health and healthcare costs [5,6,7,8]. Thus, predicting the disease is important to taking preventive action to inhibit its progression.

It is believed that DM is primarily caused by behavioral factors such as poor diet and physical inactivity. However, recent studies have demonstrated that exposure to environment-polluting chemicals (EPC) is strongly associated with the development of diabetes [9,10,11,12]. Many of the toxic effects of EPC result from aryl hydrocarbon receptor (AhR)-mediated responses and/or mitochondrial inhibition [13,14,15,16]. We previously quantified the level of human exposure to EPC using cell-based assays for AhR ligands (AhRL) and mitochondria-inhibiting substances (MIS) using 10 μL serum samples [13,14]. Our pairwise association studies revealed that AhRL was associated with components of metabolic syndrome and insulin resistance. AhRL had a positive correlation with serum insulin and homeostatic model assessment of insulin resistance (HOMA-IR) and a negative correlation with adiponectin [16]. AhRL was correlated almost linearly with the toxicity of total persistent organic pollutant (POP) mixtures present in the blood [13,14], and with MIS concentration as measured by intracellular ATP (MIS-ATP) and reactive oxygen species (MIS-ROS). Multivariate logistic regression analysis on the Korean Genome and Epidemiological Study (KoGES) data demonstrated that normal subjects with a high level of AhRL had at least a 4-fold higher risk of developing diabetes within 4 years compared with subjects having a low level of AhRL [13].

Learning patterns and predicting disease progression from large, complex, and unbalanced medical data is not easy. This complexity challenges medical researchers to apply machine learning techniques to diagnose and predict the progression of the disease [17,18]. Machine learning is a branch of artificial intelligence research that employs a variety of statistical, probabilistic, and optimization tools to learn from past data and then uses prior learning (training) to classify new data, identify new patterns, or predict novel trends [17,18,19,20,21]. A Bayesian network (BN), a machine learning technique, is a probabilistic graphical model that uses conditional independencies/dependencies between variables to build a directed acyclic graph (DAG) that visualizes the relationships between variables in a simple and compact form [20]. BNs have been widely used on complex medical data for diagnosis, prognosis, and prediction. A key advantage of the BN compared with other machine learning techniques is its interpretability, which may help uncover explanations or causative factors for symptoms or diseases [21,22]. Another important feature of the BN is that it can incorporate expert knowledge about the relationships between variables with data for construction of the BN structure [23,24]. In other words, BNs can combine expert knowledge and automatic learning from data. This is in contrast with fully data-driven machine learning techniques that may yield unreasonable results. This is one reason why BNs have been widely applied in medicine [25].

Diabetes can be delayed or prohibited by controlling the factors that affect the disease, such as diet, exercise, and EPC exposure. However, many people have difficulty maintaining their effort over the long term, because they often lack knowledge about the disease or those with diabetes/prediabetes are asymptomatic [18]. To motivate people who are at a high risk of diabetes to continue their efforts, it is important to quantify the effects of the risk factors in a way that patients can easily understand and to provide a tool that patients can easily access and that allows them to immediately see the expected consequences if they succeed in controlling the risk factors.

In this study, we aim to develop an interactive online application (app) based on a BN model that provides the predictive probabilities of prediabetes and diabetes in 4 years. The BN was trained using the data for 1531 subjects from the Ansung community-based cohort of the KoGES in 2008 [13]. This dataset contained glucose tolerance, EPC biomarkers (AhRL, MIS-ATP, MIS-ROS), and the traditional variables known to be relevant to diabetes, which were measured in 2008. It also contained glucose tolerance measured in the four-year follow up study in 2012. The online app that implements our BN prediction system provides a tool that instantly shows the prediction results given the user’s available information.

Our BN prediction used as predictors the EPC biomarkers that quantified the level of accumulation of EPC in the human body, as well as commonly used diabetes risk factors. The results from our prediction system revealed that the EPC biomarkers were dominant risk factors for diabetes progression. AhRL was the most effective predictor, and there were strong interactive effects of AhRL and MIS-ATP on future diabetes.

## 2. Materials and Methods

### 2.1. Data

The Ansung cohort of the KoGES was established to investigate the genetic and environmental etiology of common, complex diseases in Koreans. The results of the KoGES are available to the public, and a summary of the results has been published [26]. The data used in this study were downloaded from the KoGES depository with permission. In this study, we used a dataset from 1537 subjects from KoGES whose serum samples were collected for oral glucose tolerance testing (OGTT) in 2008 [13] and were used to measure AhRL, MIS-ATP, and MIS-ROS using cell-based assays [15]. These 1537 subjects were classified as having normal glucose tolerance (NGT), impaired glucose tolerance (IGT), or diabetes mellitus (DM) in both 2008 and in the 2012 follow-up study. The NGT, IGT, or DM of subjects was determined by WHO criteria based on the results of 75 g OGTT; NGT was defined as fasting plasma glucose (FPG) < 100 mg/dL and 2 h post load glucose concentrations after OGTT (2 h glucose) < 140 mg/dL; IGT was defined as 100 mg/dL ≤ FPG < 126 mg/dL and 140 mg/dL ≤ 2 h glucose < 200 mg/dL; DM was defined as FPG ≥ 126 mg/dL, or 2 h glucose ≥ 200 mg/dL, or if the subject was taking antidiabetic medication.

### 2.2. Data Processing

The raw data contained 1116 variables. However, most variables were irrelevant to diabetes and/or redundant; the irrelevant or redundant variables were discarded. In addition, variables with >70% missing values and/or having correlation coefficients of greater than 0.8 with other variables were eliminated. After this data cleaning process, glucose tolerance status at the time of data collection in 2008 (cGTOL), the three EPC biomarkers, and 18 variables that were known to be relevant to diabetes, were considered as candidate predictor variables for predicting glucose tolerance in 4 years (fGTOL). The letters “c” and “f” in cGTOL and fGTOL stand for “current” and “future”, respectively. We used GTOL in the variable names because diabetes status was determined by fasting glucose tolerance level in our study. In this paper, we will use diabetes and glucose intolerance interchangeably.

Out of the total of 1537 subjects, 6 subjects had missing values for the predictors, so they were removed from the data. The ages of the remaining 1531 subjects were between 47 and 76, and females accounted for 55.3% of the sample. The numbers (%) of subjects having NGT, IGT, and DM for cGTOL were 917 (59.8%), 242 (15.8%), and 372 (24.3%), respectively, while the number (%) of subjects having fGTOL (4 years later) of NGT, IGT, and DM were 907 (59.2%), 183 (11.9%), and 441 (28.8%), respectively. There was a slight increase in DM and a slight decrease in IGT after 4 years. Detailed descriptions of the variables are given in Table 1.

We observed that all patients with DM in 2008 remained as DM in 2012. Because such a non-variable 100% transition rate can degrade the performance of a prediction system, the 372 subjects who were in DM in 2008 were excluded from the dataset, and the data for the remaining 1159 subjects in NGT or IGT in 2008 were used to build a BN prediction model.

Table 2 summarizes the baseline characteristics of the predictor variables. It presents the mean ± standard deviation (SD) for continuous variables and the frequency (%) for discrete variables, by each group, NGT, IGT, and DM, of fGTOL. The homogeneity of each predictor variable across different groups of fGTOL was tested using the one-way analysis of variance (ANOVA) for continuous variables and chi-squared tests for discrete variables. The Tukey’s post hoc tests were conducted for predictor variables with significant ANOVA test results to find out which specific pairs of groups have different means. The means of age (Age), body mass index (BMI), waist circumference (Waist), systolic blood pressure (sysBP), high density cholesterol (HDL), and triglyceride (TG) were significantly lower in the NGT of fGTOL than in the IGT or DM. Similarly, the means of hemoglobin A1c (HbA1c), HOMA-IR, alanine aminotransferase (ALT), and aspartate aminotransferase (AST) were significantly higher in the DM than in the NGT or IGT. On the other hand, HOMA-β cell function (HOMA-β) was significantly lower in the DM than in the NGT or IGT. The serum biomarkers AhRL, MIS-ATP, and MIS-ROS had significantly different levels between the fGTOL groups. It is interesting to note that sex (Sex), smoking status (Smoke), and cGTOL had strongly significant relationships with fGTOL, while exercise (Exercise) and diabetes family history (DMFMY) had no significant relationship with fGTOL.

### 2.3. Discretization of Predictors

Because most of the continuous predictor variables in our dataset did not follow Gaussian distributions, we used a discrete BN that required the discretization (i.e., grouping or categorization) of continuous predictors, which is a process that transforms continuous variables into discrete ones. Details of the discretization criteria used in this study are given in Table 1. Waist, BMI, sysBP, HbA1c, HOMA-β, HOMA-IR, TCHL (total cholesterol), HDL, TG, ALT, and hsCRP (high-sensitivity C-reactive peptide) were discretized according to medical diagnostic criteria. AhRL, MIS-ATP, and MIS-ROS were discretized using the optimal cut-off values obtained from the receiver operating characteristic (ROC) analysis [13].

### 2.4. BN Structure

Commonly used BN structures include naïve Bayes (NB), tree augmented naïve Bayes (TAN) and general BN (GBN) [17]. NB assumes that each predictor variable is conditionally independent of the other predictors given the target variable, i.e., there is no interaction effects on the prediction of the target variable. This assumption is simple but unrealistic. TAN employs a tree structure to relax the independence assumption of NB so that each predictor variable depends on at most one other predictor, given the target variable. GBN assumes no restriction on the structure, and it does not distinguish between the target variable and the predictor variables, i.e., it considers the target variable as another predictor variable [23].

To construct the BN prediction model, also called the BN classifier, we considered TAN and GBN classifiers, which are known to outperform NB in many applications in terms of classification accuracy [23]. Moreover, TAN and GBN incorporate the interactive effects of predictors, which is more realistic in most applications.

### 2.5. Feature Selection

Feature (predictor variable) selection is the essential process of reducing the number of predictor variables to obtain a set of principal variables for building the BN classifier [19]. This process alleviates the overfitting problem caused by irrelevant or redundant variables, that may strongly bias the performance of the classifier. It also improves the interpretability of the BN structure and reduces training time. The selection of the most adequate set of features for the task of classifying objects is based on the informational theoretical concepts of information gain and mutual information (MI) [24]. In this study, we applied two feature selection methods: filter and wrapper [27,28,29]. The filter method selects features by information gain based on the entropy of each feature, and it does not depend on the BN structure. In this study, the function information.gain in R package Fselector [30] was used to implement the filter method. The wrapper method applies the prediction with a certain subset of features and evaluates the performance using cross-validation. Then, it iterates and tries a different subset of features until the optimal subset is reached. The most notable wrapper methods of feature selection are forward selection, backward selection, and stepwise selection. The wrapper method depends on the BN structure; hence, it can yield different feature subsets for TAN and GBN. In this study, we used forward selection using the function forward.search in R package FSelector.

### 2.6. BN Prediction Model

Combining the BN structure and a set of predictors, we considered six candidate BN classifiers: (i) TAN with all 22 predictors, (ii) TAN with the predictors selected from the filter method, (iii) TAN with the predictors selected from the wrapper method, (iv) GBN with all 22 predictors, (v) GBN with the predictors selected from the filter method, and (vi) GBN with the predictors selected from the wrapper method.

The predictive performances of the BN classifiers were evaluated by accuracy (%) and the area under the curve (AUC). To compute the accuracy and AUC, 10-fold stratified cross-validation was repeated 10 times. We used the R package bnlearn [31] for structure learning and parameter estimation for each BN model.

### 2.7. Online Interactive App

We integrated the proposed BN prediction system in an interactive online app called DiabetesBN [32], using the R package Shiny. In the app, the class names, normal, prediabetes, and diabetes, are used for NGT, IGT, and DM, respectively. The app shows a barplot of the predictive probabilities of normal, prediabetes, and diabetes, based on the user’s current diabetes status, EPC biomarkers, and commonly used behavioral and clinical variables. When there are non-responses, i.e., missing values, for some variables, it computes the marginal posterior probabilities given only the available information. This marginalization takes account of the errors induced by the missing values in estimation of the predictive probabilities, hence it provides more reasonable prediction results compared with the frequently used imputation methods.

DiabetesBN may return slightly different probabilities on different runs due to simulation noise because it uses cpquery function in bnlearn to compute the probabilities. The cpquery function uses Monte Carlo simulation methods for estimation. We used 5 million Monte Carlo iterations in the app, which took about 6 s to show the results and had a variation of less than 5% for different runs. More accurate estimates can be obtained by increasing the number of Monte Carlo iterations and/or using more advanced Monte Carlo algorithms, but these can increase computation time.

**Figure 1:**
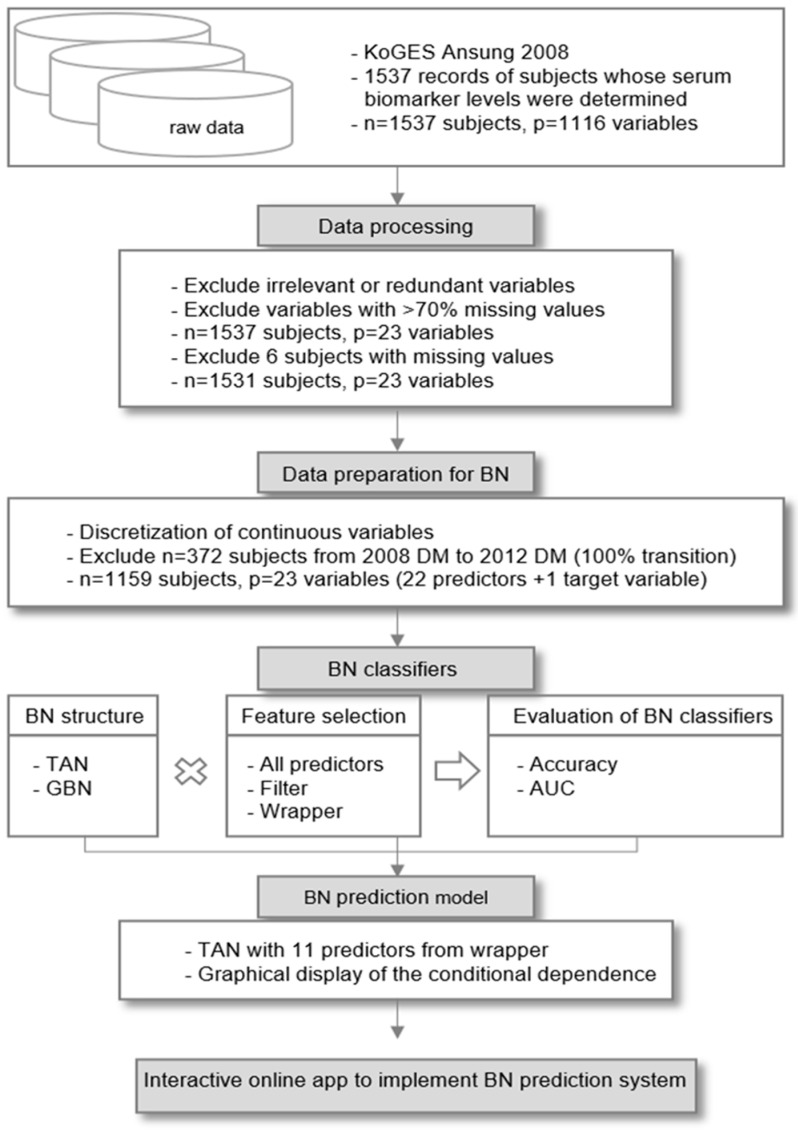
Process of developing the BN prediction app for diabetes progression.

A flow diagram of the data processing and the entire process of constructing our BN prediction system is shown in Figure 1.

## 3. Results

### 3.1. Feature Selection

**Figure 2:**
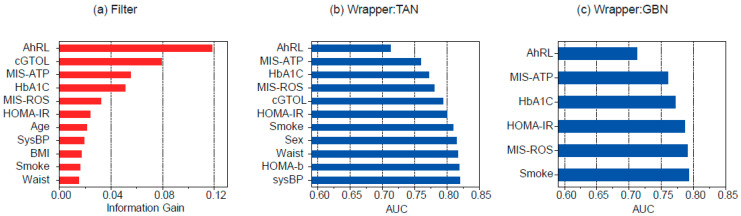
Feature selection results from (**a**) filter method, (**b**) wrapper for TAN, and (**c**) wrapper for GBN.

The variables selected using the feature selection methods in this study are shown in Figure 2. The filter method selected 11 variables based on information gain (Figure 2a). The top five variables selected from the filter method were AhRL, cGTOL, MIS-ATP, HbA1c, and MIS-ROS. Note that the filter method does not depend on the structure of the BN, so the predictors selected from the filter method can be used for both TAN and GBN. The wrapper method selected 11 variables for TAN (Figure 2b) and 6 variables for GBN (Figure 2c). The variables AhRL, MIS-ATP, HbA1c, HOMA-IR, MIS-ROS, and Smoke were selected from all the three feature selection methods.

### 3.2. BN Prediction Model

We considered six candidate BN models from combinations of two BN structures (TAN, GBN) and three sets of predictors (all 22 variables, the variables selected from the filter method, the variables selected from the wrapper method). Table 3 shows the mean ± SD of classification accuracy and AUC for the six candidate BN models. We selected the TAN with the 11 predictors selected from the wrapper method as our BN model for predicting the future development of diabetes because (i) it achieved the highest accuracy and the AUC, (ii) TAN can be considered as a compromise between NB and GBN in terms of model complexity, and (iii) it contained almost the same set of features as the filter method, while the wrapper for GBN contained only six variables. The predictors selected by the wrapper method for TAN were AhRL, cGTOL, MIS-ATP, HbA1c, MIS-ROS, HOMA-IR, Smoke, Sex, Waist, HOMA-β, and sysBP.

**Figure 3:**
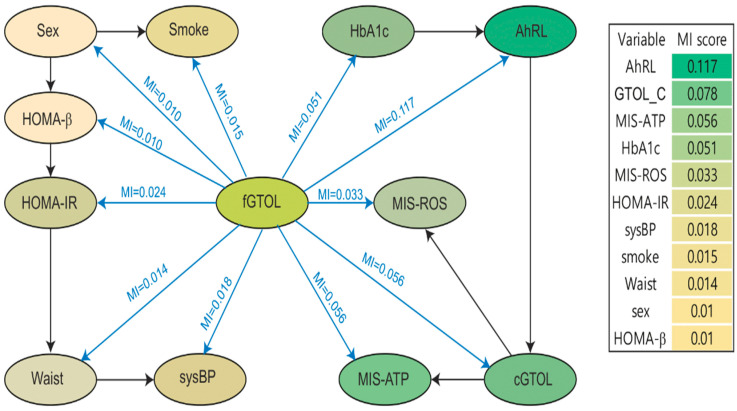
Structure of BN prediction model and mutual information (MI) between the target and each predictor node. Variables connected by edges are conditionally dependent on each other. The scores for MI between the target node fGTOL and the predictor nodes are presented on each blue edge. The variable nodes are color coded according to their MI scores. Variables in the box were sorted in descending order of MI scores.

Figure 3 is a graphical display of the probabilistic relationship between variables in our BN prediction model. Each node represents a variable. Edges (arrows connecting nodes) represent conditional dependencies; unconnected nodes represent variables that are conditionally independent of each other [33,34]. Because TAN assumes a direct relationship between each predictor and the target variable, all predictors are connected to the target (blue edges in Figure 3). The black edges in Figure 3 show the conditional dependencies between predictors. For example, given fGTOL, cGTOL is conditionally dependent on variables AhRL, MIS-ATP, and MIS-ROS, but it is conditionally independent from Sex, Smoke, HOMA-β, HOMA-IR, Waist, and sysBP.

We calculated the scores for mutual information (MI) [34] between the target variable fGTOL and the predictors. MI is a quantitative measure of the degree of interaction between each node and its parent node in a network [35]. In other words, MI (X, Y) measures the amount of information that predictor (X) provides about the target (Y). It can be computed from the marginal distributions P(X = x), P(Y = y) and the joint distribution P(X = x, Y = y) of two variables using the formula:

$$\mathrm{MI}\left(X,Y\right)=\underset{x,y}{\sum }P\left(X=x,Y=y\right)\log\frac{P\left(X=x,Y=y\right)}{P\left(X=x\right)P\left(Y=y\right)}.$$

In Figure 3, the MI scores between the target variable (fGTOL) and the predictors are presented on the edges between them. The computed MI scores ranged from 0.010 to 0.117. The top five informative variables for predicting fGTOL were AhRL (MI = 0.117), cGTOL (MI = 0.078), MIS-ATP (MI = 0.056), HbA1c (MI = 0.051), and MIS-ROS (MI = 0.033). These five variables were the serum EPC biomarkers and the well-known key indicators of diabetes. The remaining six variables were Sex, Smoke, HOMA-β, HOMA-IR, Waist, and sysBP. Interestingly, these six variables were separated from, i.e., conditionally independent from, the top five variables, given fGTOL. This conditional independence implies that the above two sets of variables had no interactive effect in predicting fGTOL.

### 3.3. Online Interactive App

**Figure 4:**
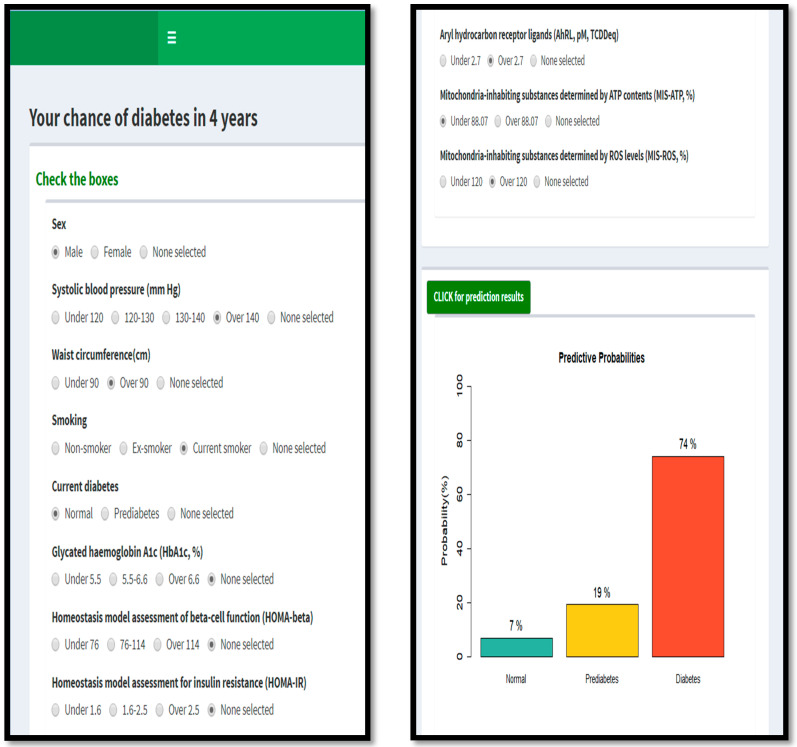
An illustration of DiabetesBN [32], the online interactive app that implements the BN prediction model for diabetes progression. This is an example of the predictive probabilities of normal (NGT), prediabetes (IGT), and diabetes (DM) in 4 years for a subject with Sex = male, Waist ≥ 90 cm, sysBP ≥ 140 mm Hg, Smoke = current, cGTOL = NGT, AhRL ≥ 2.7, MISATP < 88.07, and MISROS ≥ 120.

If one selects the values of available predictors and clicks the green button “CLICK for prediction results” in DiabetsBN, it shows the barplot of the predictive probabilities for normal (NGT), prediabetes (IGT), and diabetes (DM), along with the probabilities (%) marked above the bars. Figure 4 illustrates DiabetesBN for a subject with Sex = male, Waist ≥ 90 cm, sysBP ≥ 140 mm Hg, Smoke = current, cGTOL = NGT, AhRL ≥ 2.7, MIS-ATP < 88.07, and MIS-ROS ≥ 120. In Figure 4, his predictive probabilities for normal, prediabetes, and diabetes in 4 years are 7%, 19%, and 74%, respectively, and he has a very high chance of diabetes mellitus in 4 years. If his AhRL is low (<2.7) instead of high (≥2.7), then the predictive probabilities for normal, prediabetes, and diabetes would become approximately 35%, 19%, and 46%, respectively. This implies that if he lowers his AhRL, he could cut his chance of developing DM within 4 years by 40% and he would be more likely to stay in normal or prediabetes rather than to develop diabetes. Furthermore, if he additionally makes his Waist < 90 cm, the predictive probabilities for Normal, prediabetes, and diabetes would become approximately 60%, 19%, and 21%, respectively, and his chance of diabetes in 4 years would be substantially reduced. The above examples illustrate that by changing the values of some of the variables in the app using just a few clicks, one can simulate one’s chance of developing diabetes/prediabetes in 4 years if one changes some risk factors. This could be the driving force that will help people maintain their efforts in controlling risk factors.

Note that in the above examples, HbA1c, HOMA-IR, and HOMA-β were missing, which corresponds to the “none selected” values for these predictors in DiabetesBN. Thus, DiabetesBN provides the *marginal* predictive probabilities given only the observed predictors in these examples.

### 3.4. Effects of AhRL and MIS-ATP

The feature selection result and MI scores demonstrated that the three serum biomarkers, AhRL, MIS-ATP, and MIS-ROS, played important roles in predicting diabetes. Moreover, Figure 3 shows that these biomarkers are directly connected with cGTOL and HbA1c, which are commonly used key indicators of diabetes. Other recent studies have also revealed that these EPC biomarkers were closely related to diabetes and other metabolic diseases [13,36,37,38,39]. To see the effect of the level of human exposure to EPC on a patient’s future onset of diabetes, we investigated the effects of AhRL and MIS-ATP in our BN prediction system when cGTOL and HbA1c were adjusted for. Note that the four most important variables in our BN model were AhRL, cGTOL, MIS-ATP, and HbA1c in descending order of MI scores. Among the three EPC biomarkers, MIS-ROS was not considered because it had a relatively low MI score, and the predictive probabilities were not much different for different levels of MIS-ROS when the other four variables were given. Another reason for excluding MIS-ROS was that stratifying subjects by five variables yielded small groups having less than five subjects, from which it was difficult to obtain meaningful statistical results.

We did not adjust for variables other than cGTOL and HbA1c because of their small MI scores and small-group problems mentioned above. In addition, we observed that there were only six subjects whose HbA1c level were high (≥6.6%) among the 1159 subjects who belong to either the NGT or IGT class of cGTOL. These six outlying subjects led to very small groups in the adjustment for cGTOL and HbA1c, and hence they were excluded.

**Figure 5:**
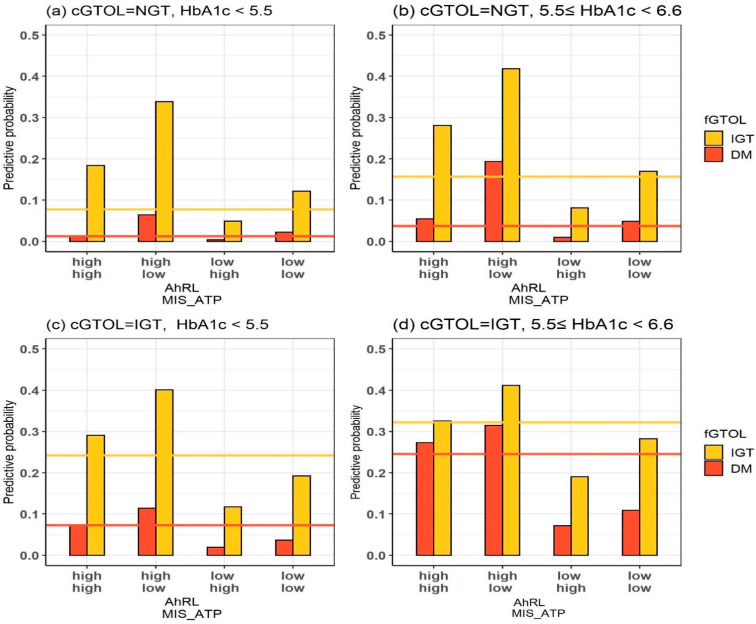
Predictive probabilities of DM (red) and IGT (yellow) for the joint levels of AhRL and MIS-ATP given cGTOL and HbA1c. The levels of AhRL are low (<2.7) and high (≥2.7), and the levels of MIS-ATP are low (<88.07) and high (≥88.07). The lines in each figure show the predictive probabilities of DM (red) and IGT (yellow), marginalized over AhRL and MIS-ATP.

Figure 5 presents the predictive probabilities of DM (red bars) and IGT (yellow bars) of fGTOL for each possible combination of AhRL and MIS-ATP levels, given cGTOL and HbA1c. The lines show the predictive probabilities of DM (red lines) and IGT (yellow lines) of fGTOL that were marginalized over AhRL and MIS-ATP, i.e., the predictive probabilities given only cGTOL and HbA1c. From the figure, one can see that the predictive probabilities varied substantially depending on the levels of AhRL and MIS-ATP. For example, given cGTOL = IGT and 5.5 ≤ HbA1c < 6.6 (Figure 5b), the predictive probability of developing DM in 4 years was 0.1818. However, when the additional information of AhRL = high (≥2.7) and MIS-ATP = low (<88.07) was given, the predictive probability of developing DM became 0.3152 (73% increase). This clearly shows that the level of exposure to EPC was a key risk factor for the future development of diabetes and that there were strong interactive effects of AhRL and MIS-ATP on diabetes progression. Moreover, for every combination of cGTOL and HbA1c levels, the levels of (AhRL, MIS-ATP) in descending order of the predictive probabilities of developing IGT/DM within 4 years were (high, low) > (high, high) > (low, low) > (low, high). The joint levels of *high AhRL* and *low MIS-ATP* resulted in the highest risk of future IGT/DM.

## 4. Discussion

We developed an interactive online app, DiabetesBN, for predicting the probabilities of normal (NGT), prediabetes (IGT), and diabetes (DM) in 4 years based on a BN, an interpretable machine learning technique. The BN was trained using the dataset obtained from the Ansung cohort study of the KoGES [13]. The serum biomarkers for the level of human exposure to EPC as well as the traditional risk factors of diabetes were used as predictor variables. After we compared the two network structures, TAN and GBN, and features selected from the filter-based and the wrapper-based methods, we selected TAN with 11 predictors from the wrapper method as our BN prediction model based on performance evaluation and practical considerations. The predictor variables in our BN model were Sex, HbA1c, HOMA-β, HOMA-IR, Smoke, Waist, sysBP, cGTOL, AhRL, MIS-ATP, and MIS-ROS.

The study on the joint effects of AhRL and MIS-ATP when cGTOL and HbA1c were adjusted for demonstrated that the above EPC biomarkers played dominant roles in diabetes progression and that they interacted. Furthermore, from the 10-fold cross-validation for performance evaluation, the accuracy and AUC of the proposed BN model were 79.43% and 0.8120, respectively, while those of the model with only the eight traditional variables (excluding the three EPC biomarkers) were 77.53% and 0.7576, respectively. It can be concluded that the additional EPC biomarkers contributed to a substantial improvement in predictive performance. This is in good agreement with our previous study results, which demonstrated that AhRL and MIS, especially MIS-ATP, were highly influential factors for the development of DM within 4 years [13].

DiabetesBN provides predictions in terms of the probability of fGTOL for all three classes—normal, prediabetes, and diabetes. The probabilities could be interpreted as weights, and they can be easily and intuitively interpreted by non-experts. Moreover, from the probabilities, one can determine the predicted class (e.g., the most probable class) and determine the uncertainties associated with the prediction. A patient with the predictive probabilities (0.1, 0.1, 0.9) for (normal, prediabetes, diabetes) would have to be treated differently from a patient with the probabilities (0.3, 0.3, 0.4), although diabetes is the most probable class for both patients. Moreover, the probabilities may allow clinicians to adopt more flexible decision rules. For instance, considering that DM is a chronic disease that affects patients’ quality of life and often calls for high health expenditure to treat its diverse complications, clinicians may declare DM as the predicted class of a patient whenever his/her probability of DM is greater than 40% and suggest more aggressive intervention to the patient.

Most previous studies on diabetes prediction or diagnosis that incorporated the effect of exposure to EPC were conducted under some assumptions about the pattern of associations. For example, the correlation coefficient measures a *linear* association between two variables, and a multiple logistic regression model assumes that there exist *linear* effects of the predictors on the log odds of the probabilities [36,37,38,39]. Correlation coefficients may miss important nonlinear associations. In the logistic regression model, it may not be easy for a non-expert to interpret the proportional effects of covariates on the odds of the probability. Moreover, unless the covariates are transformed appropriately and additional interaction terms are included as covariates, the logistic regression model detects only the linear non-interactive effects of covariates on the log odds of the probability. On the other hand, the BN, based on conditional dependencies between variables, do *not assume any specific form* of covariate effects and incorporate *interactive* effects in a natural way.

Our final dataset used for learning the BN contained about 78% of NGT, 16% IGT, and 6% DM in fGTOL. The dataset was imbalanced, like most medical datasets. However, previous studies based on a large number of imbalanced datasets have demonstrated that BN is a strong machine learning technique for an imbalanced data set [40,41,42]. Our BN prediction model also demonstrated good performance based on the AUC, which has been known to be a good metric for performance evaluation when instances are imbalanced with respect to class labels [41,42].

**Figure 6:**
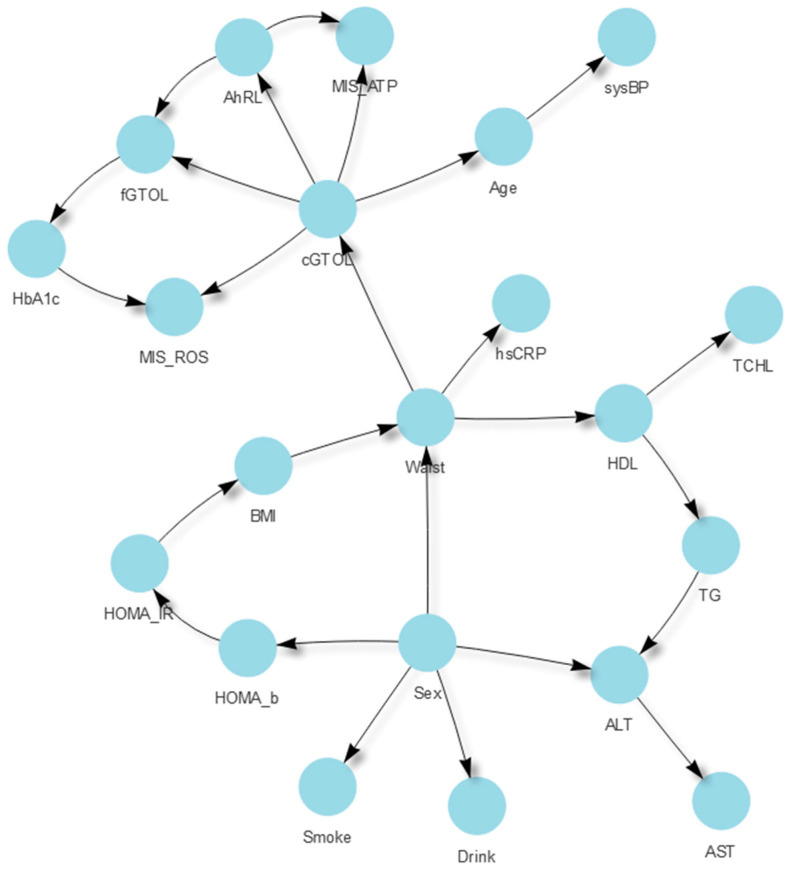
GBN structure on the variables. GBN assumed no structural constraint. Variables connected by edges are conditionally dependent on each other.

Our BN prediction model demonstrated two conditionally independent groups, given fGTOL: namely Group 1 consisting of HbA1c, cGTOL, AhRL, MIS-ATP, and MIS-ROS, and Group 2 consisting of the traditional risk factors of diabetes, Sex, Smoke, HOMA-β, HOMA-IR, Waist, and sysBP. It is notable that all variables in Group 1 have higher MI scores than those in Group 2, and that the two groups do not interact in predicting fGTOL (Figure 3). We applied a GBN that assumed no structural constraint to further investigate the relationships between the two groups. The GBN also showed a very similar separation of the two groups (Figure 6). This automatic data-driven relationship may provide helpful information in building a suitable BN for causal inference, which will be discussed next.

The cause–effect relationship is of great interest among researchers and practitioners, especially in medical support. Causal relationships can be learned from data obtained from randomized controlled experiments that allow intervention. In causal inference, intervention is to fix the values of some variables and then observe what happens to the other variables. In most practical cases, it is difficult or impossible to conduct randomized controlled experiments, and data can be obtained only from observational studies. The data used in this study were obtained from an observational study, and the directional edges in our predictive BN do not imply causality. However, the data-driven relationships between variables in the BN may provide information that can be used with expert knowledge to build a *causal* BN, in which the causalities are encoded by the directed edges of the network [25,33]. Moreover, given a causal BN, one can easily simulate the effects of intervention without the need to carry out a real-world experiment, by changing the values of some nodes that modify the distribution of other variables, called soft intervention [43,44,45]. This may be the reason why BN is popular in causal inference [25]. Our next research goal is to build a suitable causal BN for our dataset and investigate the cause–effect relationship that may help us to understand the mechanism of diabetes progression.

This study has some limitations. First, the sample size of 1159 may not be large enough to control all significant confounding factors. Second, there might have been information lost in the process of discretizing continuous variables into categorical ones. We adopted commonly used medical criteria to discretize the continuous variables in our study. However, a different number of categories and/or a different choice of thresholds could yield different prediction results. Third, we considered only TAN and GBN for candidate BN structures in this study and the structures were learned from data. The prediction model could be improved by using other BN structures and/or incorporating expert knowledge when there is useful prior information on the relationships between variables.

## 5. Conclusions

In this study, we developed an online app for the prediction of diabetes based on a BN trained from observations of the serum biomarkers of EPC exposure level and traditional risk factors for diabetes progression. The app instantly shows the predictive probabilities of diabetes (DM), prediabetes (IGT), and normal (NGT) when the user provides available current conditions. The app can also be used as a tool for simulating the possible consequences of interventions. The proposed BN model visualized the relationships between the variables in a simple and interpretable way. In terms of MI scores, AhRL was the most effective variable in the future development of diabetes, followed by cGTOL, MIS-ATP, and HbA1c. An investigation on the effects of AhRL and MIS-ATP when cGTOL and HbA1c were adjusted for also demonstrated that they were important risk factors, and they acted interactively in the prediction of diabetes. The chance of developing diabetes or prediabetes was the highest when the level of AhRL was high and the level of MIS-ATP was low, given cGTOL and HbA1c. These results support the conjecture that the accumulation of EPCs in the human body could contribute substantially to metabolic syndrome and diabetes. Further investigation needs to be conducted on the cause–effect relationships between the variables. This is our future research goal.

## Figures and Tables

**Table 1:** Description and discretization of variables.

Variable	Description	Class
fGTOL	Glucose tolerance at 4-year follow-up	NGT, IGT, DM
cGTOL	Glucose tolerance at the time of data collection	NGT, IGT, DM
Sex	Sex	Male, Female
Drink	Alcohol intake	Non-drinker, Ex-drinker, Current drinker
Smoke	Smoking status	Non-smoker, Ex-smoker, Current smoker
Exercise	Exercise	No, Yes
DMFMY	DM family history	No, Yes
Age	Age (years)	<50; 50–60; 60–70; ≥70
Waist	Waist circumference (cm)	<85; ≥85 (female), <90; ≥90 (male)
BMI	Body mass index (kg = m^2^)	<23; 23–25; 25–30; 30–35; ≥35
sysBP	Systolic blood pressure (mm Hg)	<120; 120–130; 130–140; ≥140
HbA1c	Glycated haemoglobin A1c	<5.5; 5.5–6.6; ≥6.6
HOMA-β	Homeostasis model assessment of β-cell function	<76; 76–114; ≥114
HOMA-IR	Homeostasis model assessment for insulin resistance	<1.6; 1.6–2.5; ≥2.5
TCHL	Total cholesterol (mg/dL)	<200; 200–230; ≥230
HDL	High-density lipoprotein cholesterol (mg/dL)	<40; 40–60; ≥60
TG	Triglycerides (mg/dL)	<150; 150–200; ≥200
ALT	Alanine aminotransferase (IU/L)	<40; ≥40
AST	Aspartate aminotransferase (IU/L)	<40; ≥40
hsCRP	High-sensitivity C-reactive protein (mg/L)	<1; 1–3; ≥3
AhRL	Aryl hydrocarbon receptor ligands (pM, TCDDeq)	<2.7; ≥2.7
MIS-ATP	Mitochondria-inhabiting substances determined by ATP contents (%)	<88.07; ≥88.07
MIS-ROS	Mitochondria-inhabiting substances determined by ROS levels (%)	<120; ≥120

**Table 2:** Baseline characteristics of candidate predictor variables by fGTOL, the glucose tolerance status after 4 years (2012).

		fGTOL					
		Total (N = 1159)	NGT (N = 907)	IGT (N = 183)	DM (N = 69)	Assoc *p*-Value	Post Hoc (Tukey)
Variable		Mean ± SD or N (%)					
Age		59.74 ± 8.34	59.0 ± 8.32	62.54 ± 7.98	61.6 ± 8.43	<0.001	a,b
Sex						<0.001	
	Male	499 (43.1%)	401 (55.8%)	58 (31.7%)	40 (58.0%)		
	Female	660 (56.9%)	506 (44.2%)	125 (68.3%)	29 (42.0%)		
BMI		24.13 ± 3.13	23.90 ± 3.05	25.08 ± 3.30	24.58 ± 3.19	<0.001	a
Waist		87.57 ± 8.50	86.80 ± 8.35	90.08 ± 8.26	91.09 ± 8.92	<0.001	a,b
sysBP		119.77 ± 15.74	118.34 ± 15.13	123.90 ± 16.20	127.72 ± 18.04	<0.001	a,b
HbA1c		5.50 ± 0.39	5.44 ± 0.36	5.62 ± 0.41	5.89 ± 0.42	<0.001	a,b,c
HOMA-β		112.86 ± 67.21	113.88 ± 69.07	116.88 ± 62.10	88.77 ± 49.00	0.007	b,c
HOMA-IR		2.12 ± 1.30	2.03 ± 1.27	2.50 ± 1.48	2.20 ± 0.95	<0.001	a
TCHL		191.75 ± 32.91	190.65 ± 31.97	195.57 ± 34.79	195.99 ± 38.91	0.099	
HDL		46.10 ± 10.68	46.72 ± 10.74	44.38 ± 10.61	42.39 ± 8.73	<0.001	a,b
TG		132.15 ± 80.21	124.82 ± 69.82	150.48 ± 90.67	179.80 ± 136.70	<0.001	a,b,c
ALT		22.01 ± 15.40	21.27 ± 12.97	23.08 ± 21.10	28.84 ± 23.44	<0.001	b,c
AST		24.64 ± 10.81	24.28 ± 88.99	24.61 ± 10.36	29.38 ± 24.51	<0.001	b,c
hsCRP		1.61 ± 5.08	1.63 ± 5.60	1.44 ± 2.29	3.55 ± 2.71	0.863	
DMFMY						0.332	
	No	1052 (90.8%)	827 (91.2%)	161(88.0%)	64 (92.8%)		
	Yes	107 (9.2%)	80 (8.8%)	22 (12.0%)	5 (7.2%)		
Smoke						<0.001	
	Non-	777 (67.1%)	614 (67.7%)	133 (72.7%)	30 (33.3%)		
	Ex-	194 (16.7%)	146 (16.1%)	32 (17.5%)	16 (23.2%)		
	Current	188 (16.2%)	147 (16.2%)	18 (9.8%)	23 (43.5%)		
Drink						0.041	
	Non-	587 (50.6%)	454 (50.0%)	106 (57.9%)	27 (39.1%)		
	Ex-	61 (5.3%)	45 (5.0%)	9 (4.9%)	7 (10.1%)		
	Current	511 (44.1%)	408 (45.0%)	68 (37.2%)	35 (50.7%)		
Exercise						0.216	
	No	788 (68.0%)	623 (68.7%)	115 (62.8%)	50 (72.5%)		
	Yes	371 (32.0%)	284 (31.3%)	68 (37.2%)	19 (27.5%)		
cGTOL						<0.001	
	NGT	917 (79.1%)	783 (86.3%)	109 (59.6%)	25 (36.2%)		
	IGT	242 (20.9%)	124 (13.7%)	74 (40.4%)	44 (63.8%)		
AhRL (pM)		2.03 ± 1.24	1.73 ± 1.02	2.96 ± 1.27	3.55 ± 1.42	<0.001	a,b,c
MIS-ATP (%)		91.99 ± 12.06	93.79 ± 11.94	86.53 ± 10.07	82.76 ± 9.66	<0.001	a,b
MIS-ROS (%)		112.31 ± 11.91	111.12 ± 10.69	116.2 ± 14.17	117.46 ± 16.35	<0.001	a,b

cGTOL, glucose tolerance at the time of data collection (current); fGTOL, glucose tolerance after 4 years (future); BMI, body mass index; Waist, waist circumference; sysBP, systolic blood pressure; TCHL, total cholesterol; HDL, high density cholesterol; TG, triglyceride; ALT, alanine aminotransferase; AST, aspartate aminotransferase; hsCRP, high-sensitivity C-reactive peptide; DMFMY, DM family history. “Assoc *p*-value” is the *p*-value from ANOVA or chi-square test between each row variable and fGTOL. “Post hoc (Tukey)” presents the significant difference (5% level) of each row variable between a pair of classes of fGTOL from Tukey’s post hoc test; ‘a’ between NGT and IGT of fGTOL, ‘b’ between NGT and DM, and ‘c’ between IGT and DM.

**Table 3:** Classification accuracy and the AUC of Bayesian network classifiers.

All variables		Classifier	
		TAN	GBN
All variables	Accuracy (%)	77.68 ± 2.60	76.35 ± 1.81
	AUC	0.7459 ± 0.0570	0.7868 ± 0.0528
Filter	Accuracy (%)	78.02 ± 2.64	77.77 ± 2.61
	AUC	0.7740 ± 0.0505	0.7618 ± 0.0513
Wrapper	Accuracy (%)	79.43 ± 2.94	78.23 ± 0.42
	AUC	0.8120 ± 0.0436	0.7886 ± 0.0384
